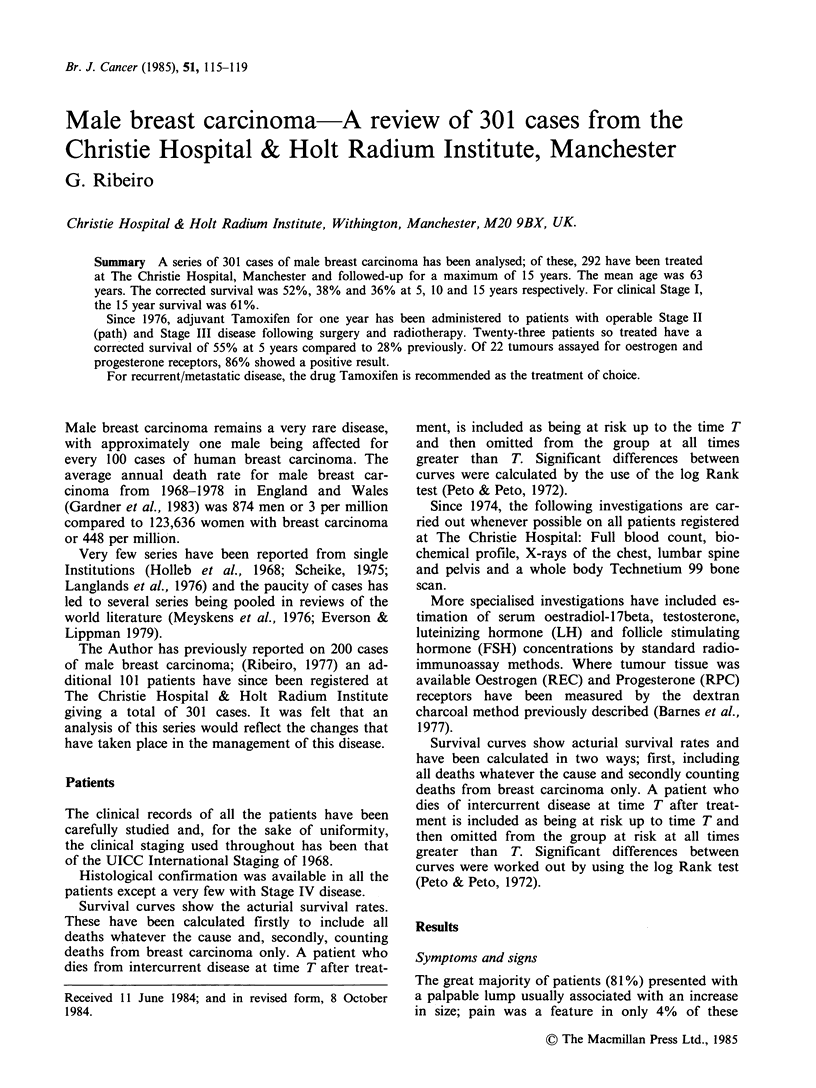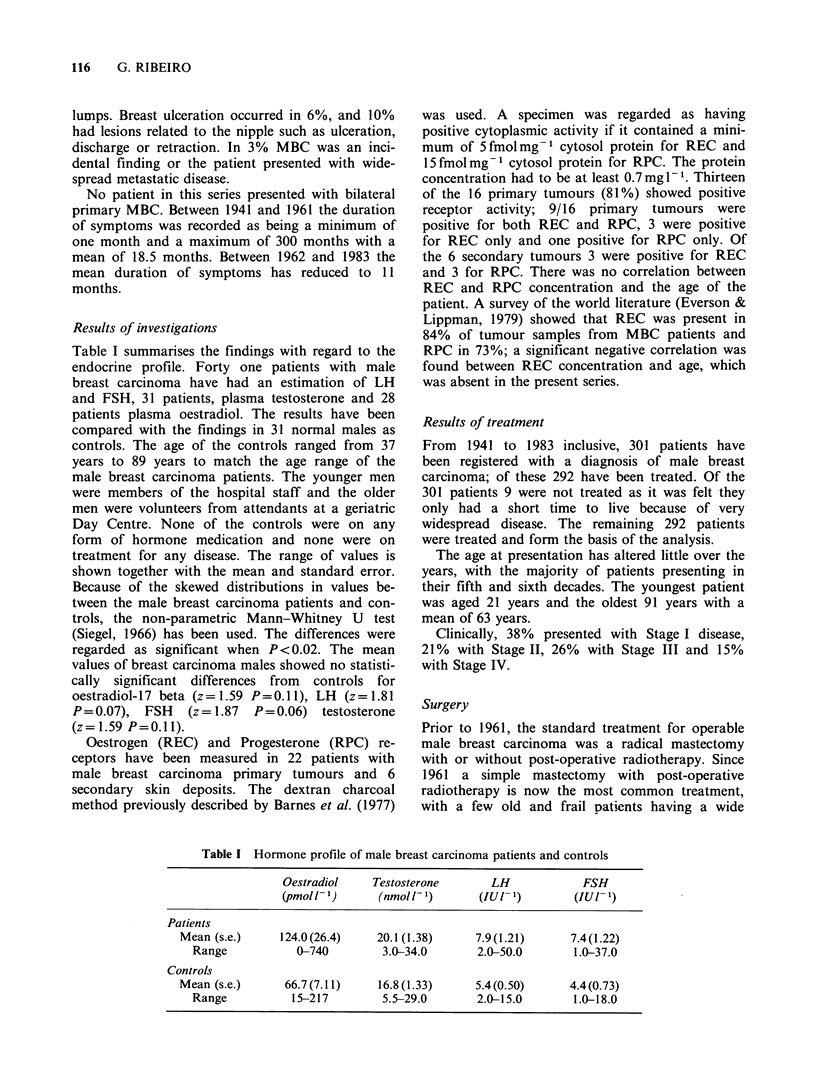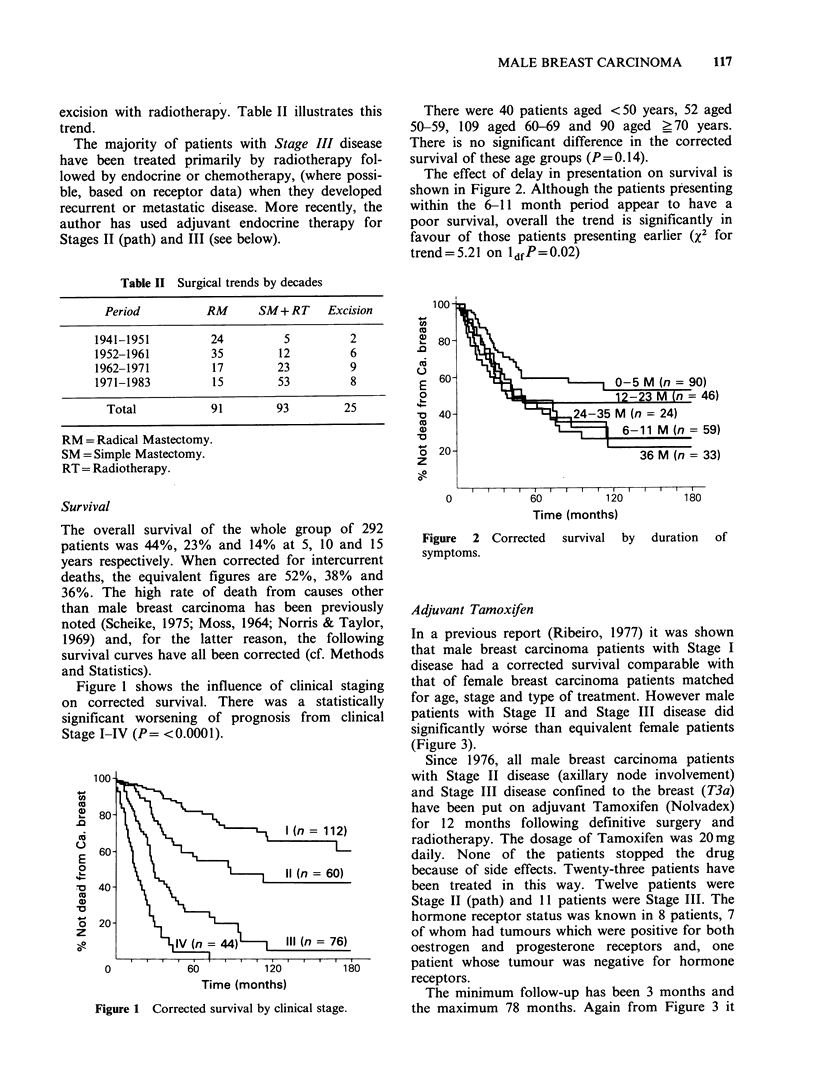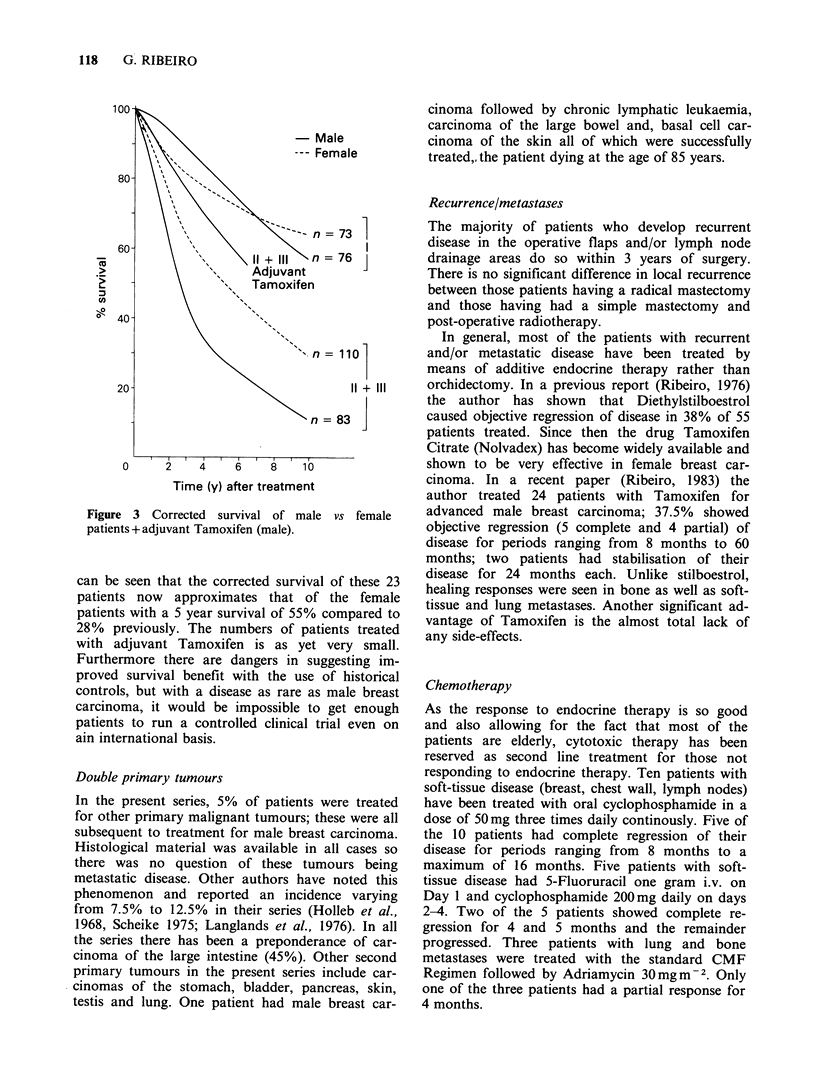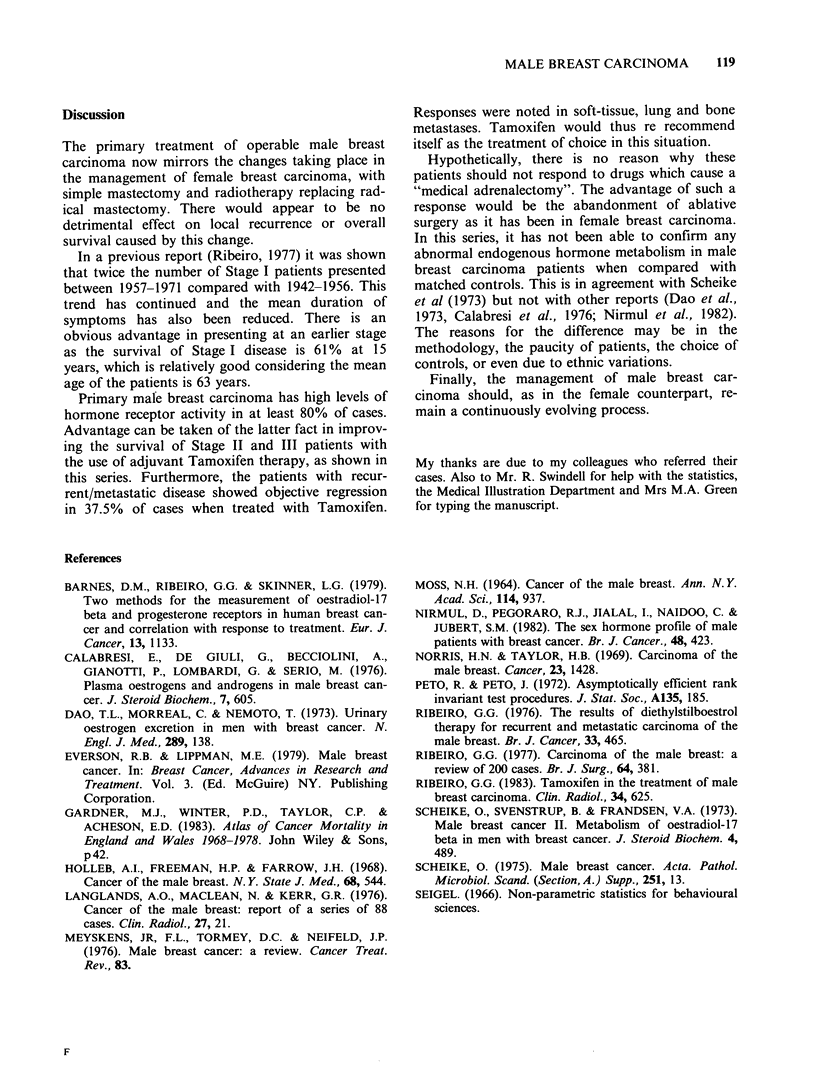# Male breast carcinoma--a review of 301 cases from the Christie Hospital & Holt Radium Institute, Manchester.

**DOI:** 10.1038/bjc.1985.16

**Published:** 1985-01

**Authors:** G. Ribeiro

## Abstract

A series of 301 cases of male breast carcinoma has been analysed; of these, 292 have been treated at The Christie Hospital, Manchester and followed-up for a maximum of 15 years. The mean age was 63 years. The corrected survival was 52%, 38% and 36% at 5, 10 and 15 years respectively. For clinical Stage I, the 15 year survival was 61%. Since 1976, adjuvant Tamoxifen for one year has been administered to patients with operable Stage II (path) and Stage III disease following surgery and radiotherapy. Twenty-three patients so treated have a corrected survival of 55% at 5 years compared to 28% previously. Of 22 tumours assayed for oestrogen and progesterone receptors, 86% showed a positive result. For recurrent/metastatic disease, the drug Tamoxifen is recommended as the treatment of choice.


Br. J. Cancer (1985), 51, 115–119

### Male breast carcinoma—A review of 301 cases from the Christie Hospital & Holt Radium Institute, Manchester

G. Ribeiro

*Christie Hospital & Holt Radium Institute, Withington, Manchester, M20 9BX, UK.*

**Summary** A series of 301 cases of male breast carcinoma has been analysed; of these, 292 have been treated at The Christie Hospital, Manchester and followed-up for a maximum of 15 years. The mean age was 63 years. The corrected survival was 52%, 38% and 36% at 5, 10 and 15 years respectively. For clinical Stage I, the 15 year survival was 61%.

Since 1976, adjuvant Tamoxifen for one year has been administered to patients with operable Stage II (path) and Stage III disease following surgery and radiotherapy. Twenty-three patients so treated have a corrected survival of 55% at 5 years compared to 28% previously. Of 22 tumours assayed for oestrogen and progesterone receptors, 86% showed a positive result.

For recurrent/metastatic disease, the drug Tamoxifen is recommended as the treatment of choice.

Male breast carcinoma remains a very rare disease, with approximately one male being affected for every 100 cases of human breast carcinoma. The average annual death rate for male breast carcinoma from 1968–1978 in England and Wales (Gardner *et al.,* 1983) was 874 men or 3 per million compared to 123,636 women with breast carcinoma or 448 per million.

Very few series have been reported from single Institutions (Holleb *et al.,* 1968; Scheike, 1975; Langlands *et al.,* 1976) and the paucity of cases has led to several series being pooled in reviews of the world literature (Meyskens *et al.,* 1976; Everson & Lippman 1979).

The Author has previously reported on 200 cases of male breast carcinoma; (Ribeiro, 1977) an additional 101 patients have since been registered at The Christie Hospital & Holt Radium Institute giving a total of 301 cases. It was felt that an analysis of this series would reflect the changes that have taken place in the management of this disease.

### Patients

The clinical records of all the patients have been carefully studied and, for the sake of uniformity, the clinical staging used throughout has been that of the UICC International Staging of 1968.

Histological confirmation was available in all the patients except a very few with Stage IV disease.

Survival curves show the acturial survival rates. These have been calculated firstly to include all deaths whatever the cause and, secondly, counting deaths from breast carcinoma only. A patient who dies from intercurrent disease at time $T$ after treatment, is included as being at risk up to the time $T$ and then omitted from the group at all times greater than $T$. Significant differences between curves were calculated by the use of the log Rank test (Peto & Peto, 1972).

Received 11 June 1984; and in revised form, 8 October 1984.

Since 1974, the following investigations are carried out whenever possible on all patients registered at The Christie Hospital: Full blood count, biochemical profile, X-rays of the chest, lumbar spine and pelvis and a whole body Technetium 99 bone scan.

More specialised investigations have included estimation of serum oestradiol-17beta, testosterone, luteinizing hormone (LH) and follicle stimulating hormone (FSH) concentrations by standard radioimmunoassay methods. Where tumour tissue was available Oestrogen (REC) and Progesterone (RPC) receptors have been measured by the dextran charcoal method previously described (Barnes *et al.,* 1977).

Survival curves show acturial survival rates and have been calculated in two ways; first, including all deaths whatever the cause and secondly counting deaths from breast carcinoma only. A patient who dies of intercurrent disease at time $T$ after treatment is included as being at risk up to time $T$ and then omitted from the group at risk at all times greater than $T$. Significant differences between curves were worked out by using the log Rank test (Peto & Peto, 1972).

### Results

### *Symptoms and signs*

The great majority of patients (81%) presented with a palpable lump usually associated with an increase in size; pain was a feature in only 4% of these

© The Macmillan Press Ltd., 1985

lumps. Breast ulceration occurred in 6%, and 10% had lesions related to the nipple such as ulceration, discharge or retraction. In 3% MBC was an incidental finding or the patient presented with widespread metastatic disease.

No patient in this series presented with bilateral primary MBC. Between 1941 and 1961 the duration of symptoms was recorded as being a minimum of one month and a maximum of 300 months with a mean of 18.5 months. Between 1962 and 1983 the mean duration of symptoms has reduced to 11 months.

*Results of investigations*

Table I summarises the findings with regard to the endocrine profile. Forty one patients with male breast carcinoma have had an estimation of LH and FSH, 31 patients, plasma testosterone and 28 patients plasma oestradiol. The results have been compared with the findings in 31 normal males as controls. The age of the controls ranged from 37 years to 89 years to match the age range of the male breast carcinoma patients. The younger men were members of the hospital staff and the older men were volunteers from attendants at a geriatric Day Centre. None of the controls were on any form of hormone medication and none were on treatment for any disease. The range of values is shown together with the mean and standard error. Because of the skewed distributions in values between the male breast carcinoma patients and controls, the non-parametric Mann–Whitney U test (Siegel, 1966) has been used. The differences were regarded as significant when $P<0.02$. The mean values of breast carcinoma males showed no statistically significant differences from controls for oestradiol-17 beta ($z=1.59$ $P=0.11$), LH ($z=1.81$ $P=0.07$), FSH ($z=1.87$ $P=0.06$) testosterone ($z=1.59$ $P=0.11$).

Oestrogen (REC) and Progesterone (RPC) receptors have been measured in 22 patients with male breast carcinoma primary tumours and 6 secondary skin deposits. The dextran charcoal method previously described by Barnes *et al.* (1977) was used. A specimen was regarded as having positive cytoplasmic activity if it contained a minimum of 5 fmol mg$^{-1}$ cytosol protein for REC and 15 fmol mg$^{-1}$ cytosol protein for RPC. The protein concentration had to be at least 0.7 mg l$^{-1}$. Thirteen of the 16 primary tumours (81%) showed positive receptor activity; 9/16 primary tumours were positive for both REC and RPC, 3 were positive for REC only and one positive for RPC only. Of the 6 secondary tumours 3 were positive for REC and 3 for RPC. There was no correlation between REC and RPC concentration and the age of the patient. A survey of the world literature (Everson & Lippman, 1979) showed that REC was present in 84% of tumour samples from MBC patients and RPC in 73%; a significant negative correlation was found between REC concentration and age, which was absent in the present series.

*Results of treatment*

From 1941 to 1983 inclusive, 301 patients have been registered with a diagnosis of male breast carcinoma; of these 292 have been treated. Of the 301 patients 9 were not treated as it was felt they only had a short time to live because of very widespread disease. The remaining 292 patients were treated and form the basis of the analysis.

The age at presentation has altered little over the years, with the majority of patients presenting in their fifth and sixth decades. The youngest patient was aged 21 years and the oldest 91 years with a mean of 63 years.

Clinically, 38% presented with Stage I disease, 21% with Stage II, 26% with Stage III and 15% with Stage IV.

*Surgery*

Prior to 1961, the standard treatment for operable male breast carcinoma was a radical mastectomy with or without post-operative radiotherapy. Since 1961 a simple mastectomy with post-operative radiotherapy is now the most common treatment, with a few old and frail patients having a wide

**Table I** Hormone profile of male breast carcinoma patients and controls

| | *Oestradiol* (*pmol* $l^{-1}$) | *Testosterone* (*nmol* $l^{-1}$) | *LH* (*IU* $l^{-1}$) | *FSH* (*IU* $l^{-1}$) |
|---|---|---|---|---|
| *Patients* | | | | |
| Mean (s.e.) | 124.0 (26.4) | 20.1 (1.38) | 7.9 (1.21) | 7.4 (1.22) |
| Range | 0–740 | 3.0–34.0 | 2.0–50.0 | 1.0–37.0 |
| *Controls* | | | | |
| Mean (s.e.) | 66.7 (7.11) | 16.8 (1.33) | 5.4 (0.50) | 4.4 (0.73) |
| Range | 15–217 | 5.5–29.0 | 2.0–15.0 | 1.0–18.0 |

excision with radiotherapy. Table II illustrates this trend.

The majority of patients with *Stage III* disease have been treated primarily by radiotherapy followed by endocrine or chemotherapy, (where possible, based on receptor data) when they developed recurrent or metastatic disease. More recently, the author has used adjuvant endocrine therapy for Stages II (path) and III (see below).

**Table II** Surgical trends by decades

| *Period* | *RM* | *SM+RT* | *Excision* |
|---|---|---|---|
| 1941–1951 | 24 | 5 | 2 |
| 1952–1961 | 35 | 12 | 6 |
| 1962–1971 | 17 | 23 | 9 |
| 1971–1983 | 15 | 53 | 8 |
| Total | 91 | 93 | 25 |

RM = Radical Mastectomy.
SM = Simple Mastectomy.
RT = Radiotherapy.

*Survival*

The overall survival of the whole group of 292 patients was 44%, 23% and 14% at 5, 10 and 15 years respectively. When corrected for intercurrent deaths, the equivalent figures are 52%, 38% and 36%. The high rate of death from causes other than male breast carcinoma has been previously noted (Scheike, 1975; Moss, 1964; Norris & Taylor, 1969) and, for the latter reason, the following survival curves have all been corrected (cf. Methods and Statistics).

Figure 1 shows the influence of clinical staging on corrected survival. There was a statistically significant worsening of prognosis from clinical Stage I–IV ($P = <0.0001$).

**Figure 1** Corrected survival by clinical stage.

There were 40 patients aged <50 years, 52 aged 50–59, 109 aged 60–69 and 90 aged ≧70 years. There is no significant difference in the corrected survival of these age groups ($P=0.14$).

The effect of delay in presentation on survival is shown in Figure 2. Although the patients presenting within the 6–11 month period appear to have a poor survival, overall the trend is significantly in favour of those patients presenting earlier ($\chi^2$ for trend $=5.21$ on $1_{df}P=0.02$)

**Figure 2** Corrected survival by duration of symptoms.

*Adjuvant Tamoxifen*

In a previous report (Ribeiro, 1977) it was shown that male breast carcinoma patients with Stage I disease had a corrected survival comparable with that of female breast carcinoma patients matched for age, stage and type of treatment. However male patients with Stage II and Stage III disease did significantly worse than equivalent female patients (Figure 3).

Since 1976, all male breast carcinoma patients with Stage II disease (axillary node involvement) and Stage III disease confined to the breast (*T3a*) have been put on adjuvant Tamoxifen (Nolvadex) for 12 months following definitive surgery and radiotherapy. The dosage of Tamoxifen was 20 mg daily. None of the patients stopped the drug because of side effects. Twenty-three patients have been treated in this way. Twelve patients were Stage II (path) and 11 patients were Stage III. The hormone receptor status was known in 8 patients, 7 of whom had tumours which were positive for both oestrogen and progesterone receptors and, one patient whose tumour was negative for hormone receptors.

The minimum follow-up has been 3 months and the maximum 78 months. Again from Figure 3 it

**Figure 3** Corrected survival of male *vs* female patients+adjuvant Tamoxifen (male).

can be seen that the corrected survival of these 23 patients now approximates that of the female patients with a 5 year survival of 55% compared to 28% previously. The numbers of patients treated with adjuvant Tamoxifen is as yet very small. Furthermore there are dangers in suggesting improved survival benefit with the use of historical controls, but with a disease as rare as male breast carcinoma, it would be impossible to get enough patients to run a controlled clinical trial even on ain international basis.

*Double primary tumours*

In the present series, 5% of patients were treated for other primary malignant tumours; these were all subsequent to treatment for male breast carcinoma. Histological material was available in all cases so there was no question of these tumours being metastatic disease. Other authors have noted this phenomenon and reported an incidence varying from 7.5% to 12.5% in their series (Holleb *et al.*, 1968, Scheike 1975; Langlands *et al.*, 1976). In all the series there has been a preponderance of carcinoma of the large intestine (45%). Other second primary tumours in the present series include carcinomas of the stomach, bladder, pancreas, skin, testis and lung. One patient had male breast carcinoma followed by chronic lymphatic leukaemia, carcinoma of the large bowel and, basal cell carcinoma of the skin all of which were successfully treated,, the patient dying at the age of 85 years.

*Recurrence/metastases*

The majority of patients who develop recurrent disease in the operative flaps and/or lymph node drainage areas do so within 3 years of surgery. There is no significant difference in local recurrence between those patients having a radical mastectomy and those having had a simple mastectomy and post-operative radiotherapy.

In general, most of the patients with recurrent and/or metastatic disease have been treated by means of additive endocrine therapy rather than orchidectomy. In a previous report (Ribeiro, 1976) the author has shown that Diethylstilboestrol caused objective regression of disease in 38% of 55 patients treated. Since then the drug Tamoxifen Citrate (Nolvadex) has become widely available and shown to be very effective in female breast carcinoma. In a recent paper (Ribeiro, 1983) the author treated 24 patients with Tamoxifen for advanced male breast carcinoma; 37.5% showed objective regression (5 complete and 4 partial) of disease for periods ranging from 8 months to 60 months; two patients had stabilisation of their disease for 24 months each. Unlike stilboestrol, healing responses were seen in bone as well as soft-tissue and lung metastases. Another significant advantage of Tamoxifen is the almost total lack of any side-effects.

*Chemotherapy*

As the response to endocrine therapy is so good and also allowing for the fact that most of the patients are elderly, cytotoxic therapy has been reserved as second line treatment for those not responding to endocrine therapy. Ten patients with soft-tissue disease (breast, chest wall, lymph nodes) have been treated with oral cyclophosphamide in a dose of 50 mg three times daily continously. Five of the 10 patients had complete regression of their disease for periods ranging from 8 months to a maximum of 16 months. Five patients with soft-tissue disease had 5-Fluoruracil one gram i.v. on Day 1 and cyclophosphamide 200 mg daily on days 2–4. Two of the 5 patients showed complete regression for 4 and 5 months and the remainder progressed. Three patients with lung and bone metastases were treated with the standard CMF Regimen followed by Adriamycin 30 mg m$^{-2}$. Only one of the three patients had a partial response for 4 months.

### Discussion

The primary treatment of operable male breast carcinoma now mirrors the changes taking place in the management of female breast carcinoma, with simple mastectomy and radiotherapy replacing radical mastectomy. There would appear to be no detrimental effect on local recurrence or overall survival caused by this change.

In a previous report (Ribeiro, 1977) it was shown that twice the number of Stage I patients presented between 1957–1971 compared with 1942–1956. This trend has continued and the mean duration of symptoms has also been reduced. There is an obvious advantage in presenting at an earlier stage as the survival of Stage I disease is 61% at 15 years, which is relatively good considering the mean age of the patients is 63 years.

Primary male breast carcinoma has high levels of hormone receptor activity in at least 80% of cases. Advantage can be taken of the latter fact in improving the survival of Stage II and III patients with the use of adjuvant Tamoxifen therapy, as shown in this series. Furthermore, the patients with recurrent/metastatic disease showed objective regression in 37.5% of cases when treated with Tamoxifen. Responses were noted in soft-tissue, lung and bone metastases. Tamoxifen would thus re recommend itself as the treatment of choice in this situation.

Hypothetically, there is no reason why these patients should not respond to drugs which cause a "medical adrenalectomy". The advantage of such a response would be the abandonment of ablative surgery as it has been in female breast carcinoma. In this series, it has not been able to confirm any abnormal endogenous hormone metabolism in male breast carcinoma patients when compared with matched controls. This is in agreement with Scheike *et al* (1973) but not with other reports (Dao *et al.*, 1973, Calabresi *et al.*, 1976; Nirmul *et al.*, 1982). The reasons for the difference may be in the methodology, the paucity of patients, the choice of controls, or even due to ethnic variations.

Finally, the management of male breast carcinoma should, as in the female counterpart, remain a continuously evolving process.

My thanks are due to my colleagues who referred their cases. Also to Mr. R. Swindell for help with the statistics, the Medical Illustration Department and Mrs M.A. Green for typing the manuscript.

### References

BARNES, D.M., RIBEIRO, G.G. & SKINNER, L.G. (1979). Two methods for the measurement of oestradiol-17 beta and progesterone receptors in human breast cancer and correlation with response to treatment. *Eur. J. Cancer*, **13**, 1133.

CALABRESI, E., DE GIULI, G., BECCIOLINI, A., GIANOTTI, P., LOMBARDI, G. & SERIO, M. (1976). Plasma oestrogens and androgens in male breast cancer. *J. Steroid Biochem.*, **7**, 605.

DAO, T.L., MORREAL, C. & NEMOTO, T. (1973). Urinary oestrogen excretion in men with breast cancer. *N. Engl. J. Med.*, **289**, 138.

EVERSON, R.B. & LIPPMAN, M.E. (1979). Male breast cancer. In: *Breast Cancer, Advances in Research and Treatment*. Vol. 3. (Ed. McGuire) NY. Publishing Corporation.

GARDNER, M.J., WINTER, P.D., TAYLOR, C.P. & ACHESON, E.D. (1983). *Atlas of Cancer Mortality in England and Wales 1968–1978*. John Wiley & Sons, p42.

HOLLEB, A.I., FREEMAN, H.P. & FARROW, J.H. (1968). Cancer of the male breast. *N.Y. State J. Med.*, **68**, 544.

LANGLANDS, A.O., MACLEAN, N. & KERR, G.R. (1976). Cancer of the male breast: report of a series of 88 cases. *Clin. Radiol.*, **27**, 21.

MEYSKENS, JR, F.L., TORMEY, D.C. & NEIFELD, J.P. (1976). Male breast cancer: a review. *Cancer Treat. Rev.*, **83.**

MOSS, N.H. (1964). Cancer of the male breast. *Ann. N.Y. Acad. Sci.*, **114**, 937.

NIRMUL, D., PEGORARO, R.J., JIALAL, I., NAIDOO, C. & JUBERT, S.M. (1982). The sex hormone profile of male patients with breast cancer. *Br. J. Cancer.*, **48**, 423.

NORRIS, H.N. & TAYLOR, H.B. (1969). Carcinoma of the male breast. *Cancer*, **23**, 1428.

PETO, R. & PETO, J. (1972). Asymptotically efficient rank invariant test procedures. *J. Stat. Soc.*, **A135**, 185.

RIBEIRO, G.G. (1976). The results of diethylstilboestrol therapy for recurrent and metastatic carcinoma of the male breast. *Br. J. Cancer*, **33**, 465.

RIBEIRO, G.G. (1977). Carcinoma of the male breast: a review of 200 cases. *Br. J. Surg.*, **64**, 381.

RIBEIRO, G.G. (1983). Tamoxifen in the treatment of male breast carcinoma. *Clin. Radiol.*, **34**, 625.

SCHEIKE, O., SVENSTRUP, B. & FRANDSEN, V.A. (1973). Male breast cancer II. Metabolism of oestradiol-17 beta in men with breast cancer. *J. Steroid Biochem.* **4**, 489.

SCHEIKE, O. (1975). Male breast cancer. *Acta. Pathol. Microbiol. Scand. (Section, A.) Supp.*, **251**, 13.

SEIGEL. (1966). Non-parametric statistics for behavioural sciences.